# Lifetime Deletion of Skeletal Muscle Keap1 Attenuates Aging-Induced Cardiac Dysfunction via an Nrf2–Antioxidant Mechanism

**DOI:** 10.3390/antiox14121491

**Published:** 2025-12-12

**Authors:** Kanika Sharma, Sarah Pribil Pardun, Neha Dhyani, Irving H. Zucker, Bipin G. Nair, Sudarslal Sadasivan Nair, Vikas Kumar, Lie Gao

**Affiliations:** 1 Amrita School of Biotechnology, Amrita Vishwa Vidyapeetham, Amritapuri Campus, Kollam 690525, Kerala, India; kasharma@unmc.edu (K.S.); bipin@am.amrita.edu (B.G.N.); 2 Multiomics Mass Spectrometry and Proteomics Core Facility, University of Nebraska Medical Center, Omaha, NE 68198, USA; 3 Department of Anesthesiology, University of Nebraska Medical Center, Omaha, NE 68198, USA; spribil@unmc.edu; 4 Department of Cellular & Integrative Physiology, University of Nebraska Medical Center, Omaha, NE 68198, USA; ndhyani@unmc.edu (N.D.); izucker@unmc.edu (I.H.Z.); 5 Department of Biochemistry and Molecular Biotechnology, UMass Chan Medical School, Shrewsbury, MA 01545, USA; vikas.kumar5@umassmed.edu

**Keywords:** aging, nuclear factor erythroid 2-related factor, cardiac dysfunction, Keap1 knockout, interorgan antioxidant crosstalk, myocardial proteomics

## Abstract

**Background**: Aging elevates reactive oxygen species (ROS) and weakens antioxidant defenses, contributing to cardiac dysfunction. The objective of this study was to determine whether sustained activation of skeletal muscle (SkM) Nrf2 preserves cardiac function during aging and to explore the underlying mechanisms, focusing on myocardial antioxidant pathways. **Methods**: Tamoxifen-induced SkM-specific Keap1 knockout male mice (iMS-*Keap1^flox/flox^*; SkM-Nrf2 overexpression) were divided into young wild-type (Y-WT), aged wild-type (A-WT), and aged knockout (A-KO) groups. Cardiac performance was evaluated by echocardiography and invasive hemodynamics. Myocardial proteomics identified differentially expressed proteins (DEPs) and enriched biological pathways. **Results**: Compared with Y-WT, A-WT mice showed impaired left ventricular function, including reduced ejection fraction, prolonged isovolumic relaxation time, blunted inotropic response to dobutamine, and elevated Tau index. These age-related deficits were partially reversed in A-KO mice. Proteomic analysis revealed 561 DEPs between A-WT and Y-WT, and 741 DEPs between A-KO and A-WT, enriched in calcium signaling, Nrf2-mediated oxidative stress response, oxidative phosphorylation, ROS detoxification, and cardiac-specific processes, such as hypertrophy, conduction, and dilated cardiomyopathy. **Conclusions**: Lifelong SkM-Nrf2 activation strengthens myocardial antioxidant capacity and alleviates age-related cardiac dysfunction. These data support an antioxidant crosstalk between skeletal muscle and the heart, highlighting a potential therapeutic target for aging-associated heart failure.

## 1. Introduction

Aging is a complex biological process characterized by progressive decline in organ function and increased susceptibility to chronic diseases, including cardiovascular dysfunction and heart failure [1]. Among the molecular mechanisms driving aging, oxidative stress and redox imbalance play a central role, promoting cellular senescence and organ deterioration [2,3]. Recent studies further highlight the importance of mitochondrial regulators, including mitochondrial sirtuins, in maintaining redox homeostasis and moderating age-associated physiological decline [4]. The transcription factor nuclear factor erythroid 2-related factor 2 (Nrf2), a master regulator of antioxidant responses, has garnered significant attention for its role in mitigating oxidative damage and preserving cellular health [5,6]. Under normal conditions, Nrf2 activity is tightly regulated by Kelch-like ECH-associated protein 1 (Keap1), which facilitates the ubiquitination and degradation of Nrf2. Disruption of this regulation, such as through silencing or suppression of Keap1, has emerged as a promising therapeutic strategy to counteract oxidative stress-related pathologies, including aging-associated cardiac dysfunction [7]. Consistent with this concept, growing evidence suggests that mitochondrial dysfunction, altered bioenergetics, and oxidative stress remain central drivers of cardiovascular disease in aging populations [8].

Skeletal muscle (SkM) is not only a key effector organ for systemic energy metabolism and body movement but also a significant contributor to interorgan communication. However, the mechanisms underlying this interorgan crosstalk and the extent of its influence remain poorly understood. Nrf2 signaling has emerged as a pivotal regulator of antioxidant defense and detoxification pathways. While recognized for its role in protecting SkM against oxidative stress, recent studies have revealed its broader impact on other organs, particularly the heart [9]. The upregulation of Nrf2 signaling in SkM has been extensively studied for its role in enhancing the muscle’s resilience to oxidative stress, thereby contributing to improved exercise performance and protection against muscle-wasting conditions [10]. However, emerging evidence suggests that the effects of Nrf2 upregulation extend beyond SkM, impacting the function and health of the heart [11]. In parallel, endothelial senescence and vascular aging processes, heavily influenced by oxidative stress and impaired Nrf2 signaling, are now recognized as major contributors to cardiovascular decline in the elderly [12], further underscoring the importance of systemic redox regulation in aging.

The role of interorgan communication between SkM and the heart is pivotal in mediating exercise-induced benefits. During physical activity, SkM releases extracellular vesicles (EVs), which contain antioxidant enzymes and multiple signaling molecules, such as myokines, growth factors, and microRNAs, that influence cardiac function and remodeling [13,14]. These SkM-derived EVs enhance antioxidant capacity, promote anti-inflammatory effects, upregulate myocardial metabolism, and improve cardiac remodeling, contributing to overall cardiovascular benefits induced by exercise. Simultaneously, the heart adapts to the increased demands of exercise by improving cardiac output and releasing cardiokines that support SkM perfusion, nutrient delivery, and waste removal. This bidirectional communication ensures systemic coordination, optimizing energy utilization and recovery while also fostering long-term benefits, like enhanced cardiovascular fitness, reduced risk of metabolic diseases, and improved quality of life [13]. The complex interactions among different tissues in the body are key to understanding the intricacies of aging and disease. Understanding the interaction between SkM and the myocardium in the context of Nrf2 signaling has important implications for both normal adaptation and disease states. Further research into this crosstalk could reveal new therapeutic approaches for cardiovascular diseases and muscle disorders, underscoring the importance of considering the systemic impact of tissue-specific interventions that target Nrf2 signaling.

The current study investigated the role of sustained SkM-specific Nrf2 overexpression on aging-induced cardiac dysfunction. Our focus was to elucidate the impact of Nrf2 activation in SkM on the heart, a phenomenon with profound implications for overall health and longevity. Utilizing the tamoxifen-induced, SkM-specific, Keap1-deletion transgenic mouse model, iMS-*Keap1^flox/flox^*, created in our laboratory [15], we hypothesized that chronic activation of SkM Nrf2 improves cardiac function in aging. By integrating echocardiographic and hemodynamic assessments with a comprehensive Label-Free Quantification (LFQ) analysis of the myocardium, we aimed to unravel the molecular pathways underpinning the cardioprotective effects of SkM Nrf2 modulation as a strategy for preserving cardiovascular health during aging.

## 2. Materials and Methods

All animal procedures adhered strictly to the guidelines outlined in the National Institutes of Health Guide for the Care and Use of Laboratory Animals and complied with Animal Research: Reporting of In Vivo Experiments (ARRIVE) guidelines. Approval was granted by the Animal Care and Use Committee of the University of Nebraska Medical Center (UNMC-IACUC Protocol #22-017-05).

### 2.1. Animal Model

In the present study, we utilized thirty-one male 12-week-old iMS-*Keap1^flox/flox^* mice, a tamoxifen-induced, skeletal muscle-specific, Keap1 knockout model generated and bred in our laboratory, as previously described [15]. The mice were divided into three groups: young wild-type (Y-WT; 23.84 ± 2.01 g; n = 11), aged wild-type (A-WT; 29.01 ± 2.53 g; n = 11), and aged Keap1 knockout (A-KO; 31.56 ± 2.17 g; n = 9). The sample size was based on our prior studies [16,17] using mouse models and echocardiographic/hemodynamic outcomes, which demonstrated that group sizes of 8–14 are sufficient to detect physiologically meaningful differences in mouse left ventricular function in both normal and chronic heart failure conditions. Mice in the A-KO group received intraperitoneal injection (ip) of tamoxifen (2 mg/0.2 mL sunflower oil/day × 5 days, Sigma-Aldrich, St. Louis, MO, USA; Cat. No. T5648) at 3 months of age. Mice in the Y-WT and A-WT groups received equivalent volumes of sunflower oil (vehicle) at the same age. At the age of 6 months in the young group and 27 months in the aged groups, cardiac function was evaluated by echocardiography and left ventricular (LV) hemodynamics, followed by a comprehensive LFQ analysis of the LV myocardium. SkM-specific Keap1 gene knockout was determined by PCR analysis of genomic DNA from the gastrocnemius (Gas), livers, and hearts. The sequences of primers were 5′-GAG TCC ACA GTG TGT GGC C-3′ and 5′-GAG TCA CCG TAA GCC TGG TC-3′. The thermal cycler parameters were (1) 95 °C—5 min; (2) 95 °C—30 s; (3) 62 °C—30 s; (4) 72 °C—2 min; cycle steps 2–4 × 34 times; (5) 72 °C—20 min; and (6) 4 °C—until analysis.

### 2.2. Cardiac Function Evaluation

#### 2.2.1. Echocardiography

Echocardiograms were carried out under isoflurane anesthesia at the age of 6 months for the young group and 27 months for the aged groups. The anesthesia process began with induction using 2–3% isoflurane delivered in 100% oxygen via an induction chamber. Once the mouse was unresponsive to stimuli and exhibited regular, slow breathing, it was transferred to a heated imaging platform equipped with nose-cone delivery of 0.5–1% isoflurane. Anesthetic depth was continuously monitored by respiratory rate, chest excursions, the absence of withdrawal reflex, and stable heart rate on the ECG leads of the imaging system. The isoflurane concentration was adjusted within the 0.5–1% range as needed to maintain a stable physiological state throughout the echocardiography session. By using a Vevo 3100 ultrasound system (VisualSonics, Toronto, ON, Canada) with a 40-MHz probe, 2D B-mode images were acquired in the long- and short-parasternal axis, while M-Mode images were acquired at the level of the left ventricular papillary muscles. Left ventricular volumes and diameters were measured. Ejection fraction (EF) was calculated by a standard formula: [(LVEDV × LVESV)/LVEDV] × 100. Fractional shortening (FS) was calculated as [(LVEDD × LVESD)/LVEDD] × 100. The echocardiographer was blinded to the animal groups. In addition to baseline cardiac function, echocardiography was also used to determine cardiac reserve. Cardiac reserve was evaluated by the change in cardiac output, EF, and FS in response to i.p. injection of the β1 agonist, dobutamine (2 mg/kg), taken at 3 min following injection.

#### 2.2.2. Left Ventricular Hemodynamics Measurement

Under 2% isoflurane anesthesia, mice were placed on a metal heating pad in the supine position. The right common carotid artery was dissected, and a pressure transducer (SPR-1000; Millar Instruments, Houston, TX, USA) was advanced into the ascending aorta and left ventricle for the measurement of blood pressure, left ventricular pressure, the first derivative of left ventricular pressure (dP/dt), and heart rate (HR). All signals were processed by a Power-Lab data acquisition system (Model 8S) and analyzed using LabChart 8 software with the blood pressure module (ADInstruments, Inc., Colorado Springs, CO, USA).

### 2.3. Western Blot Assay, Proteomics, and Bioinformatics Analysis

#### 2.3.1. Tissue Harvesting and Processing

Following evaluation of cardiac function, mice were euthanized through CO_2_ inhalation. The left ventricle and soleus muscle were swiftly excised and then finely minced using sterile surgical scissors. The minced tissue was transferred into pre-chilled microcentrifuge tubes containing RIPA buffer (50 mM Tris-HCl, 195 mM NaCl, 2 mM EDTA, 1% NP-40, 0.1% SDS) supplemented with 1% protease inhibitor cocktail (Abcam, Cambridge, UK, ab65621). The tissue samples were then homogenized on ice using a tissue homogenizer to ensure complete lysis and protein extraction. Following homogenization, the lysates were centrifuged at 12,000 rpm for 15 min at 4 °C to remove cellular debris. The resulting supernatants, containing the protein extracts, were carefully transferred to fresh microcentrifuge tubes and stored at −80 °C until further analysis.

#### 2.3.2. Western Blot Assay

The protein concentration of the extract was measured using a protein assay kit (Pierce; Rockford, IL, USA) and then adjusted to equal volume in all samples with 2× 4% SDS sample buffer. The samples were heated to 95 °C for 5 min and then loaded on a precast polyacrylamide gel (NW00105BOX, Invitrogen, Waltham, MA, USA), along with 2 μL of Invitrogen MagicMark™ Western Standard (Invitrogen, Waltham, MA, USA) and 4 μL of Bio Rad Precision Plus Protein Dual Color Standards (Bio Rad, Hercules, CA, USA) in separate wells (15 μg in 15 μL/each sample). Electrophoresis was performed using a Mini Gel Tank and Blot Module Set (NW2000, Invitrogen, Waltham, MA, USA), initially at 70 V, for approximately 15 min, and then at 120 V, until the desired separation was reached. The fractionated protein on the gel was electrically transferred onto a nitrocellulose membrane by employing Thermo Fischer Scientific iBlot2 (IB21001, Thermo Fisher Scientific, Waltham, MA, USA) with the preset program P0 (20 V for 1 min, then 23 V for 4 min, followed by 25 V for 2 min). The membranes were gently rinsed with Milli-Q water and then stained with Ponceau S for 5 min to visualize total protein. Once ideal bands of total protein were obtained, Ponceau S on the membrane was removed by rinsing it in 10 mL of 1× phosphate-buffered saline–Tween 20 solution (PBST) for 5 min. The membranes were then blocked for 30 min in 5% milk in 1× PBST at room temperature; this was followed by two 5 min washes in 10 mL of 1× PBST at room temperature. The membranes were then incubated in primary antibodies (Anti-Glutathione S-transferase A2 (GSTA2) antibody-ab232833, Anti-NAD(P)H dehydrogenase (quinone 1) (NQO1) antibody-ab80588, and Anti-Nuclear Factor Erythroid 2-related Factor 2 (Nrf2) antibody-ab137550) in 4% BSA overnight at 4 °C. The following day, the membranes were washed three times for 5 min in 10 mL of 1× PBST; this was followed by incubation in anti-rabbit IgG horseradish peroxidase secondary antibody (1:5000) in 5% milk in 1× PBST for 30 min at room temperature. After this, the membranes were again washed three times for 5 min in 10 mL of 1× PBST. The membranes were developed in chemiluminescence detection reagent (SuperSignal West Dura; Thermo Scientific, Waltham, MA, USA). The blots on the membrane were visualized using G:Box Syngene and Genesys (SYNGENE, Cambridge, UK). The intensity of the bands was quantified using ImageJ software (NIH, Bethesda, MD, USA; https://imagej.net/, accessed on 4 April 2025) and normalized for loading using Ponceau S staining. The Western blot was completed by a technician blinded to the groups.

#### 2.3.3. Mass Spectrometry-Based Proteomics

Protein concentration was measured using a Pierce protein assay kit (Pierce; Rockford, IL, USA). For global proteomic analysis, 100 µg of protein from six biological replicates per group was prepared and processed. The samples were reduced with 10 mM DTT at 55 °C and alkylated with 50 mM iodoacetamide at room temperature. Detergents were removed via chloroform/methanol extraction, and the resulting protein pellet was re-suspended in 50 mM ammonium bicarbonate. Digestion with MS-grade trypsin (Pierce) was carried out overnight at 37 °C. Peptides were cleaned using PepClean C18 spin columns (Thermo Scientific™, Waltham, MA, USA) and re-suspended in 2% acetonitrile (ACN) and 0.1% formic acid (FA). Subsequently, 1 µg of each sample was loaded onto a trap column (Acclaim PepMap 100 75 µm × 2 cm C18 LC Columns; Thermo Scientific, Waltham, MA, USA) and separated using a Thermo RSLC Ultimate 3000 system (Thermo Scientific, Waltham, MA, USA) on a Thermo Easy-Spray PepMap RSLC C18 75 µm × 50 cm column (Thermo Scientific, Waltham, MA, USA). Peptides were eluted with a step gradient of solvent B (0.1% FA in 80% ACN) and analyzed by a Thermo Orbitrap Exploris 480 mass spectrometer in data-dependent acquisition mode. The acquisition parameters included a survey full-scan MS with a resolution of 120,000, isolation of ions with charge states 2–6, and fragmentation using HCD (Thermo Scientific, Waltham, MA, USA) with 35% normalized collision energy. Protein identification was performed by searching the MS/MS data against the Mus musculus protein database using the SequestHT (Matrix Science, Boston, MA, USA) search engine. Quantitative analysis was conducted using the global expression workflow in Proteome Discoverer 3.0 (Thermo Scientific™, Waltham, MA, USA), with protein expression fold changes represented as Log2FC between the young and aging Nrf2-Keap WT and Nrf2-Keap KO groups.

#### 2.3.4. Differential Proteomic and Pathway Enrichment Analyses

Proteins identified via mass spectrometry underwent quantification to discern variations in expression levels between experimental and control conditions using Thermo Proteome Discover 3.0 software. Furthermore, gene enrichment analysis of the identified differentially regulated proteins was conducted to identify affected functions, pathways, and networks. This analysis was performed using Ingenuity Pathway Analysis (IPA) from Ingenuity Systems (Mountain View, CA, USA). For differential expression analysis summary and IPA pathway analysis, the cut-off *p*-value used was ≤0.05, and the absolute fold change was >1.5. For volcano plots, the cut-off for adding gene names to differentially expressed proteins was an absolute Log2 fold change greater than 1 and a *p*-value of 0.05 or less.

### 2.4. Statistical Analysis

All data for cardiac function measurement and Western blot analysis are expressed as means ± SD. The Shapiro–Wilk test was used to assess whether the data were normally distributed. If normally distributed, a two-way ANOVA followed by the Newman–Keuls test for post hoc analysis was used when multiple comparisons were made among the Y-WT, A-WT, and A-KO groups. *p* < 0.05 is statistically significant.

## 3. Results

### 3.1. SkM-Specific Deletion of the Keap1 Gene

**Figure 1:**
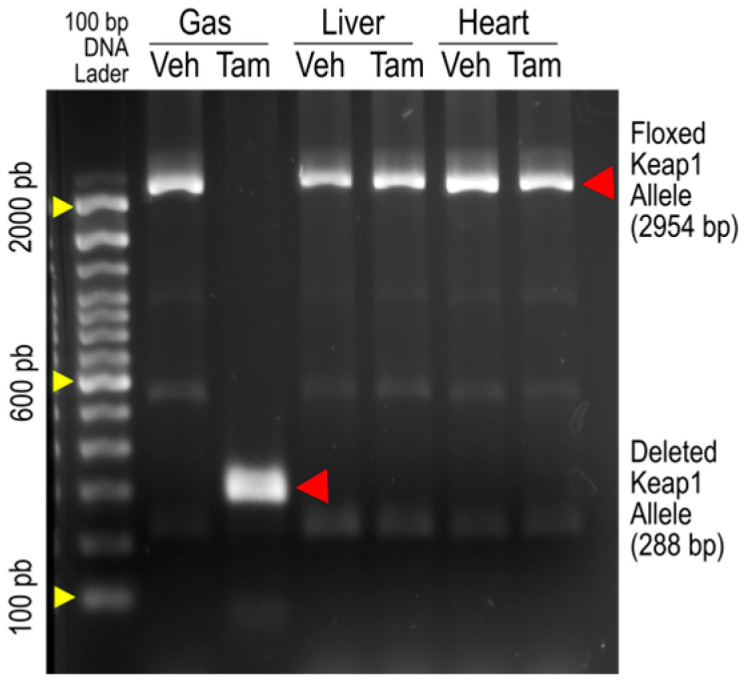
PCR analysis of genomic DNA from the gastrocnemius (Gas), liver, and heart of iMS-*Keap1^flox/flox^* mice revealed intact floxed Keap1 allele (2954 bp) and deleted Keap1 allele segments (288 bp).

Figure 1 is an original image of agarose gel electrophoresis showing PCR amplification of Keap1 alleles from the gastrocnemius (Gas), livers, and hearts of iMS-*Keap1^flox/flox^* mice treated with vehicle (Veh) or Tamoxifen (Tam). The 2954-bp bands represent the intact Keap1 alleles, while the 288-bp band represents an allele whose Exons 2 and 3 were deleted. The amplification of the deleted allele was found only in SkM of the Tam-treated mouse, suggesting that there are no off-target effects in the liver or heart by this Keap1 knockout strategy, as reported previously by our laboratory [15] and others [18].

### 3.2. Protein Expression of GSTA2, NQO1, and Nrf2

**Figure 2:**
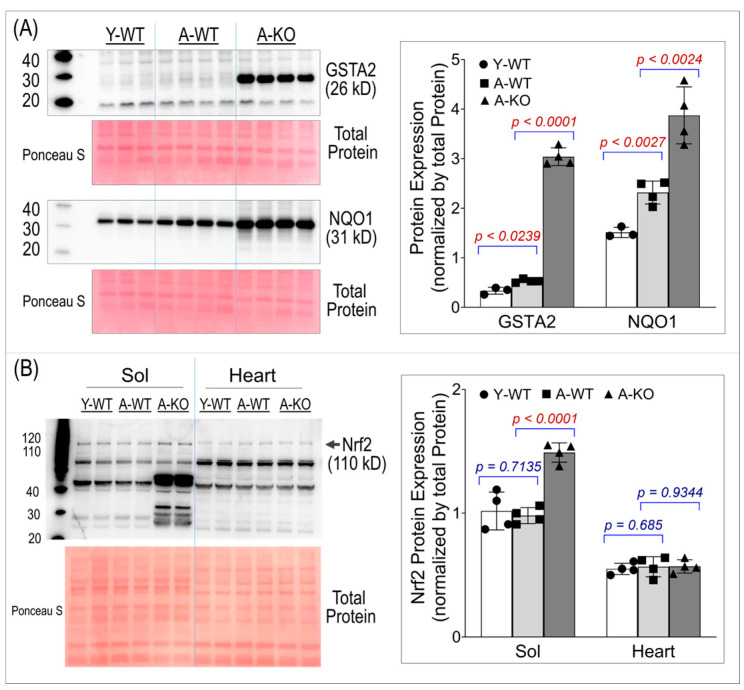
Western blot analyses of soleus muscle and hearts for GSTA2 and NQO1 (**A**) and Nrf2 (**B**). Data are presented as mean ± SD, with n = 4 per group.

To ensure conditional activation of the Nrf2–antioxidant system in SkM, we assessed the protein expression of two key Nrf2 downstream antioxidant enzymes, Glutathione S-transferase Alpha 2 (GSTA2) and NAD(P)H quinone oxidoreductase 1 (NQO1). Panel A of Figure 2 shows significant overexpression of both enzymes in the SkM of the aged mice, suggesting that high levels of antioxidant proteins last throughout life in these mice. Panel B of Figure 2 shows Nrf2 protein expression in SkM and the heart, demonstrating that Nrf2 was upregulated only in the SkM of the aged KO mice. The band used for quantification in this study is the ~110 kD band, which corresponds to the post-translationally modified, activated form of Nrf2 [19,20,21]. Notably, an additional band at ~66 kD, matching the predicted molecular weight based on the amino acid sequence, was also detected and exhibited a more pronounced increase in the aged KO soleus muscle. This lower-molecular-weight band likely represents a non-activated form of Nrf2. Because the functional downstream antioxidant response is driven by activated Nrf2, the ~66 kD band was not used for quantitative analyses.

### 3.3. Baseline Echocardiography

**Figure 3:**
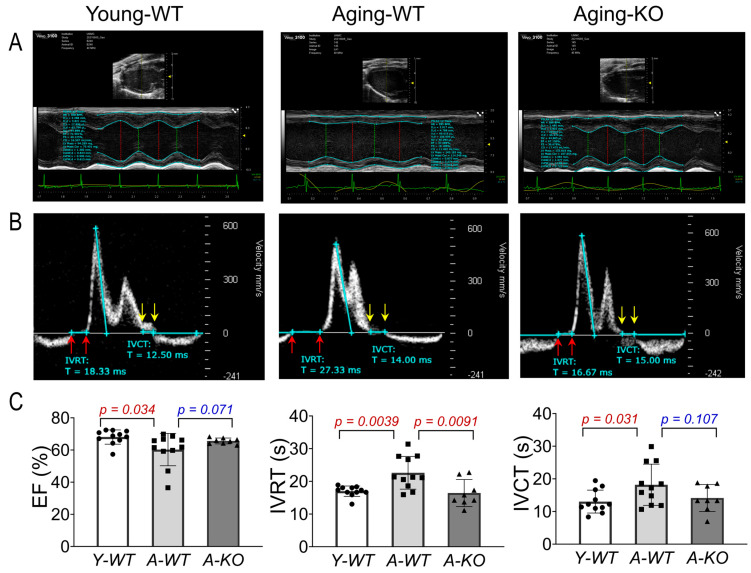
Echocardiographic analyses of left ventricular function in Y-WT, A-WT, and A-KO mice: (**A**) parasternal long axis view images in M-mode; (**B**) pulsed wave (PW) Doppler mode images; (**C**) mean data of EF, IVRT, and IVCT. Data are presented as mean ± SD, with n = 8–11 per group.

Using high-frequency ultrasound and a comprehensive echocardiographic analysis, we assessed left ventricular function of the mice in the Y-WT, A-WT, and A-KO groups. As can be seen in Figure 3, the A-WT mice exhibited a modest but significant decline in EF as compared to Y-WT (A-WT 60.3 ± 10.1% vs. Y-WT 68.1 ± 4.4%; *p* = 0.034, n = 11/group). However, in the A-KO mice, this aging-related decline in EF was attenuated (66.9 ± 4.3%, *p* = 0.071 vs. A-WT, n = 8–11/group). Importantly, isovolumetric relaxation time (IVRT) was clearly and significantly prolonged in the A-WT mice as compared with Y-WT (A-WT 22.6 ± 5.0 vs. Y-WT 16.9 ± 1.58, *p* = 0.0039; n = 11/group). Notably, lifelong overexpression of SkM-Nrf2 completely restored the IVRT, similar to that of the young mice. These data strongly suggest that lifetime muscle overexpression of Nrf2 prevents aging-induced cardiac diastolic dysfunction, providing a novel strategy to ameliorate age-associated heart failure with reduced ejection fraction (HFrEF). On the other hand, isovolumetric contraction time (IVCT) was significantly prolonged in the A-WT mice as compared with the Y-WT mice and tended to be shortened in the A-KO mice. However, this difference did not reach statistical significance.

### 3.4. Dobutamine Stress Echocardiography

**Figure 4:**
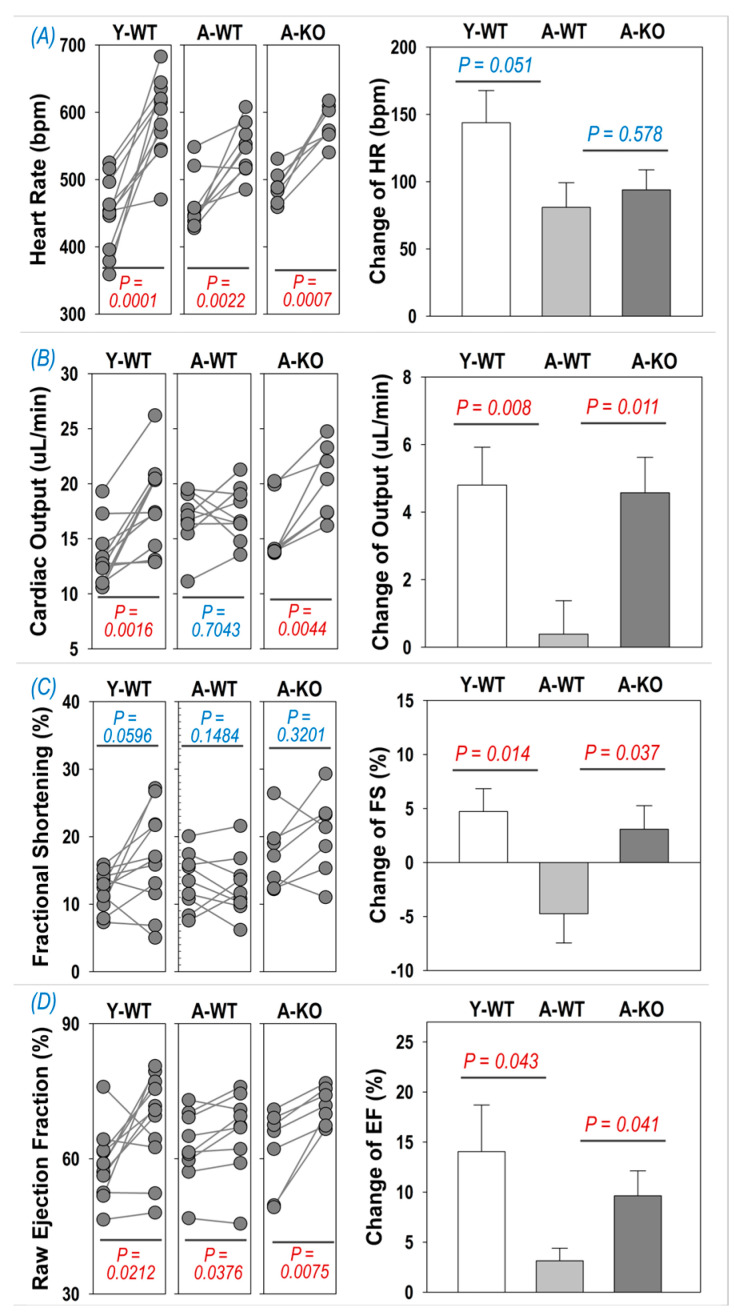
Dobutamine-induced increase in heart rate (**A**), cardiac output (**B**), fractional shortening (**C**), and ejection fraction (**D**) in Y-WT, A-WT, and A-KO mice. The left subpanels depict raw values of each mouse before and after dobutamine treatment, while the right subpanels show the change in heart rate, cardiac output, fractional shortening, and ejection fraction. Data are presented as mean ± SD, with n = 7–8 per group.

To determine if cardiac reserve is altered in aged mice following SkM upregulation of Nrf2, we examined the echocardiographic response to i.p. injection of dobutamine. As can be seen in Figure 4A, dobutamine evoked a significant increase in HR in all groups (left subpanel), whereas the changes were not significantly different between the A-WT and A-KO mice. On the other hand, the inotropic responses to dobutamine were depressed in the A-WT mice but significantly improved in the A-KO mice, as shown in the right subpanels of Figure 4B–D for the change in cardiac output, fractional shortening, and ejection fraction.

### 3.5. Left Ventricular Hemodynamics

**Figure 5:**
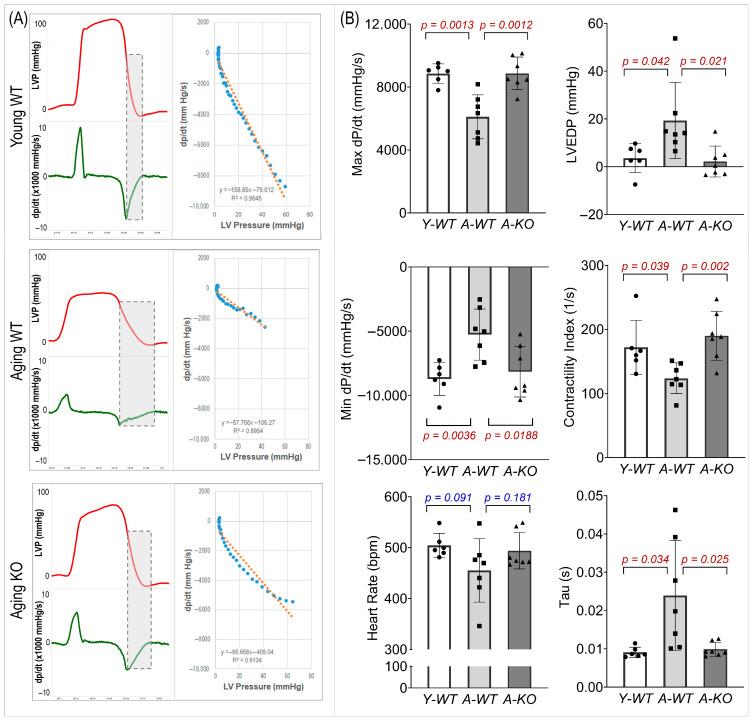
Representative hemodynamic recordings (**A**) and calculated functional parameters (**B**) of the left ventricle of mice in the Y-WT, A-WT, and A-KO groups. Data are presented as mean ± SD, with n = 6–7 per group.

We evaluated LV systolic and diastolic function (Figure 5). In the A-WT mice, LVP and maximal/minimal dP/dt were reduced. However, these measures were significantly improved in the A-KO mice. In addition, the A-WT mice exhibited a decreased LV contractility index and increased LV end-diastolic pressure (LVEDP) and Tau, whereas these changes were attenuated in the A-KO mice.

### 3.6. Mass Spectrometry-Based Global Expression Analysis

**Figure 6:**
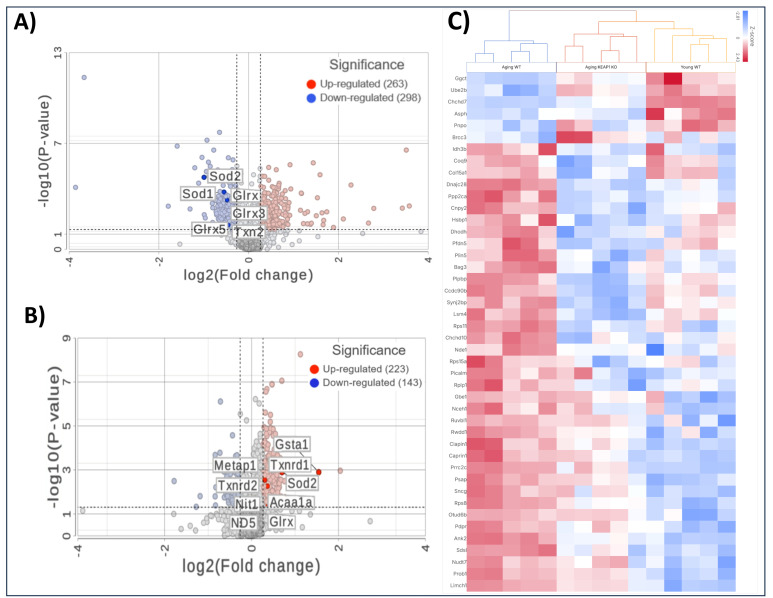
Mass spectrometry-based proteomic analyses of heart tissues. Volcano plots illustrating differentially expressed proteins comparing A-WT to Y-WT mice (**A**) and comparing A-KO to A-WT mice (**B**). Red dots represent significantly upregulated proteins, and blue dots represent significantly downregulated proteins based on fold change and the *p*-value threshold. Key proteins are labeled in each plot. (**C**) Heatmap of selected differentially expressed proteins across the Y-WT, A-WT, and A-KO groups, highlighting distinct clustering patterns and pathway-specific changes.

Comparing the A-WT mice with the Y-WT mice in the proteomics LFQ study, we observed profound alterations in cardiac protein expression profiles, indicative of age-related molecular remodeling. As can be seen in Figure 6A, 561 proteins were significantly differentially expressed, including 298 downregulated and 263 upregulated proteins. Several antioxidant proteins were found to be downregulated in the A-WT mice compared to their younger counterparts. Among these downregulated antioxidant proteins were Superoxide Dismutase 1 (SOD1), Superoxide Dismutase 2 (SOD2), Thioredoxin 2 (Txn2), and Glutathione S-Transferase kappa 1 (Gstk1), as shown in the volcano plot. In addition to antioxidant proteins, the proteomics LFQ analysis identified several other proteins implicated in various biological processes associated with aging, including mitochondrial proteins Frataxin (Fxn), Mimitin (Ndufaf2), and structural proteins, such as Collagen (Col15a1) and Muscleblind (Mbnl1), which were downregulated in the A-WT mice.

Importantly, in the A-KO mice compared to their WT counterparts, a notable trend emerged related to antioxidant proteins—they were predominantly upregulated, as shown in Figure 6B. Among these upregulated antioxidant proteins were SOD1, Txn2, and Gstk1. This marked upregulation of antioxidant proteins in the A-KO group suggests a protective mechanism against age-related oxidative stress. In addition to the upregulation of antioxidant proteins, the LFQ analysis unveiled alterations in the expression of several other proteins implicated in various biological processes associated with aging. Notably, mitochondrial proteins such as Frataxin (Fxn), Superoxide Dismutase 2 (SOD2), and Mimitin (Ndufaf2), along with structural proteins like Collagen (Col15a1) and Muscleblind (Mbnl1), exhibited differential expression patterns, with a tendency towards downregulation in the A-KO mice as compared to the A-WT mice. Overall, the comparison between the A-KO and A-WT mice highlights the contrasting expression level of antioxidant proteins, suggesting molecular mechanisms for the improved cardiac function in aging mice with SkM-Keap1 deletion.

### 3.7. Pathway Enrichment Analysis

**Figure 7:**
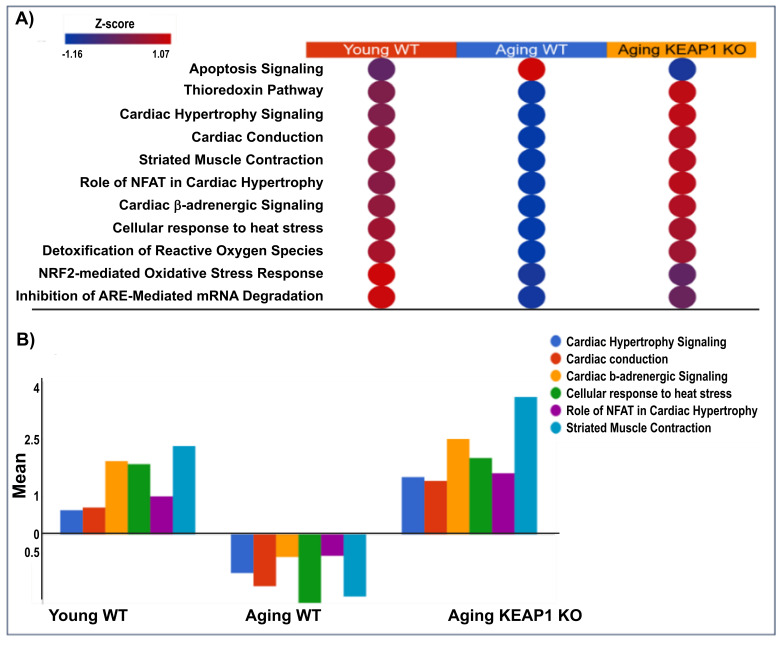
Canonical pathway analysis of redox and cardiac-related pathways in heart tissue across experimental groups. The top panel displays a heatmap (**A**) of pathway activation Z-scores, illustrating upregulation (red) and downregulation (blue) of key pathways in Y-WT, A-WT, and A-KO mice. Pathways include apoptosis signaling, thioredoxin pathway, cardiac hypertrophy signaling, cardiac conduction, striated muscle contraction, and others. The bottom panel (**B**) quantifies Z-scores for selected pathways, emphasizing shifts in pathway activity among the experimental groups.

All the differentially expressed proteins identified were subjected to canonical pathway analysis to gain insights into the potential mechanisms for the impaired cardiac function of A-WT and improved cardiac function of A-KO. As shown in Table 1, the log fold change values of antioxidant proteins highlight distinct trends in these mice. This trend underscores the role of Keap1 deletion in enhancing antioxidant defense through Nrf2-mediated pathways. Pathway analysis comparing A-WT with Y-WT and A-KO with A-WT reveals distinctive molecular pathways associated with redox homeostasis and cardiac function. In the A-WT mice, the Nrf2 pathway, known for its pivotal role in detoxifying reactive oxygen species (ROS), exhibited significant activity, reflecting the organism’s response to oxidative stress associated with aging. In contrast, in the Y-WT mice, this pathway may be reduced due to the robust antioxidant defenses characteristic of youth. In the A-KO mice, dysregulation of redox homeostasis is evident. Moreover, pathway analysis uncovers alterations in cardiac-related pathways, such as cardiac hypertrophy and cardiac conduction, particularly pronounced in aging mice of both genotypes. Figure 7 shows the reversible effects of redox and cardiac pathways in the Y-WT, A-WT, and A-KO mice. Notably, in the Y-WT mice, there was a robust and balanced relationship between redox signaling and cardiac pathways, indicative of a healthy physiological status. In the A-WT mice, a distinct pattern emerged, characterized by dysregulated redox signaling and cardiac pathway alterations. While Nrf2 activation typically reduces oxidative stress, persistent and unregulated Nrf2 signaling, as seen in Keap1 KO mice, may lead to a state of reductive stress [22], which paradoxically disrupts redox-sensitive pathways and contributes to cardiac dysfunction. Strikingly, Figure 7 portrays an opposing trend in the A-WT mice, where redox and cardiac pathways exhibit divergent trajectories compared to both the young WT and A-KO mice. This observation underscores the complexity of aging-associated changes, wherein A-WT mice may experience a shift in redox and cardiac pathways that differ from those observed in A-KO mice.

## 4. Discussion

The findings of this study provide compelling evidence that overexpression of SkM antioxidant proteins evoked by upregulation of Nrf2 attenuates aging-induced cardiac dysfunction and increases cardiac reserve in male mice. A-WT mice displayed hallmark features of mild cardiac dysfunction, including reduced EF, prolonged IVCT and IVRT, and increased Tau. Notably, these age-related impairments were partially attenuated in A-KO mice, suggesting that SkM Nrf2 activation exerts a cardioprotective effect. The proteomic analysis revealed significant changes in the myocardial protein profile of A-KO mice compared to A-WT mice. These findings underscore the importance of redox homeostasis in maintaining cardiac function and suggest that SkM Nrf2 overexpression influences cardiac health by modulating systemic oxidative stress. Beyond oxidative signaling, other pathways, including calcium signaling, cardiac hypertrophy signaling, and dilated cardiomyopathy signaling pathways, were significantly altered in A-KO mice. These pathways are critical for cardiac excitation–contraction coupling and structural integrity, providing a mechanistic basis for the observed improvements in cardiac diastolic function. The normalization of Tau in A-KO mice further supports the hypothesis that SkM Nrf2 activation alleviates myocardial stiffness and enhances ventricular relaxation, two key determinants of diastolic performance. The results of this study align with the well-documented cardioprotective effects of exercise, suggesting that SkM Nrf2 activation may serve as a molecular mimic of chronic exercise. By targeting oxidative stress and improving systemic redox balance, SkM Nrf2 activation appears to be a promising strategy for mitigating the deleterious effects of aging on cardiac function. Importantly, previous studies from our laboratory have clearly shown upregulation of antioxidant proteins and Nrf2 following exercise [23] or Keap1 deletion [15]. In addition, a recent study from our laboratory demonstrated that electrical pulse simulation of cultured myotubes led to upregulation of Nrf2-dependent antioxidant enzymes [24].

Another interesting and potentially important finding is that upregulation of SkM Nrf2 restores cardiac reserve as the response to acute β1 adrenergic receptor stimulation with dobutamine in A-KO mice (Figure 4). The mechanism(s) for this observation are not entirely clear, but we speculate that long-term upregulation of SkM Nrf2 and antioxidant proteins reduces sympathetic nervous outflow to the myocardium during the aging process, thus restoring β1 adrenergic signaling. In previous studies from our laboratory, we showed that deletion of Nrf2 in the rostral ventrolateral medulla of mice led to an increase in renal sympathetic outflow [25,26]. Conversely, upregulation of Nrf2 following deletion of Keap1 in the same area reduced sympathetic nerve activity in mice with chronic heart failure [17]. While we have not examined sympathetic nerve activity in aged mice, multiple studies have reported increased sympathetic outflow during the aging process in animals and humans [27,28,29,30,31].

It is important to consider the mechanism by which SkM Nrf2 and/or antioxidant proteins protect the aging heart. We and others have shown that EVs released from injured tissue are capable of transferring an antioxidant enzyme cohort to remote tissues [13,26,32,33,34,35]. Similarly, during exercise, EVs are released and travel to remote tissues [33,36]. Thus, SkM serves as a significant source of transferable antioxidant proteins that may have remote effects in multiple tissues, including the heart. Although Keap1 can interact with multiple substrates, extensive evidence indicates that Nrf2 is its predominant physiological target in redox regulation. In our model, the pronounced and sustained upregulation of canonical Nrf2-dependent antioxidant enzymes in SkM (e.g., GSTA2, NQO1), together with the enrichment in Nrf2-associated oxidative stress–response pathways in the myocardial proteome, provides strong functional evidence that Nrf2 activation is the principal driver of the observed cardiac phenotype. Thus, although additional Keap1-regulated mechanisms cannot be entirely excluded, the combined molecular and functional profiles in A-KO mice are most consistent with an Nrf2-centered mode of cardioprotection.

The impact of SkM Nrf2 and Keap1 on cardiac tissue has shed light on the crucial role of interorgan crosstalk in maintaining cardiac function. Through proteomic and bioinformatic analyses conducted in mouse models with SkM-specific Nrf2 and Keap1 KO, more than 100 proteins were found to be redox-related, some of which were related to cardiac function [15]. Mechanisms underlying cardiac dysfunction in aging have focused on the role of certain redox proteins, particularly Nrf2, in mitigating age-related cardiac pathology [37]. Building upon established mechanisms of cardiac dysfunction in aging, including oxidative stress, inflammation, and mitochondrial dysfunction [38], the current study examined the potential contribution of Nrf2-mediated antioxidant defense mechanisms in preserving cardiac function with advancing age. We sought to elucidate the role of Nrf2 in age-related changes in cardiac function. Through proteomic analyses and functional assessments, we characterized the expression levels and activity of Nrf2 and its downstream targets in aged cardiac tissue. Our findings provide valuable insights into the role of Nrf2 in modulating oxidative stress and inflammation within the aging heart, thereby offering potential avenues for therapeutic interventions aimed at preserving cardiac health during aging [9,39].

Previous research has extensively demonstrated the crucial role of Nrf2 in maintaining cellular redox balance and protecting against oxidative stress, not only within skeletal muscle but also systemically [40]. Our study builds upon this foundation by investigating the potential interplay between skeletal muscle Nrf2 and cardiac health. While past studies have primarily focused on the role of Nrf2 within skeletal muscle, our investigation extends this inquiry to examine its impact on cardiac function. By integrating proteomic profiling with functional assessments in cardiac tissue, we aimed to clarify how skeletal muscle Nrf2 activation influences myocardial health and performance. Through this approach, we sought to provide a comprehensive understanding of the systemic effect of Nrf2 on oxidative stress modulation and its implications for cardiac health during aging.

There may be multiple potential mechanisms underlying Nrf2-mediated cardioprotection, including its effects on oxidative stress, inflammation, and mitochondrial dysfunction within the heart. In the present study, we provide evidence suggesting that aging leads to alterations in cardiac function proteins and redox pathways, which are reversed by upregulating Nrf2 in SkM. Nrf2 activation is known to trigger the expression of antioxidant enzymes, such as SOD and catalase, thereby mitigating oxidative stress and reducing cellular damage [41]. In addition, Nrf2 activation suppresses inflammatory pathways by inhibiting the production of pro-inflammatory cytokines and chemokines [42]. Furthermore, Nrf2 activation has been associated with improvements in mitochondrial function, including enhanced mitochondrial biogenesis and ATP production, as well as maintenance of mitochondrial membrane potential [43]. By elucidating the changes in redox and cardiac proteins and pathways in response to SkM Nrf2 overexpression, the current study provides valuable insights into the potential mechanisms by which Nrf2 may confer cardioprotection, offering promising avenues for therapeutic interventions aimed at preserving cardiac structure and function during aging.

Although this study provides strong evidence demonstrating the beneficial effects of SkM Nrf2 activation on aging-related cardiac dysfunction, several limitations should be acknowledged. First, to avoid tamoxifen interactions with the endogenous hormonal system of female mice, we exclusively used male mice in this tamoxifen-inducible Cre-loxP model. As a result, the conclusions drawn here cannot be directly generalized to females. Future studies employing Cre systems that do not rely on estrogen receptor modulators will be necessary to determine whether skeletal muscle Nrf2 activation confers similar cardioprotective effects in female animals. On the other hand, a previous study demonstrated that tamoxifen alone had no effect on cardiac structure or function regardless of the dose used [44]. Second, although our findings highlight the beneficial impact of sustained SkM-Nrf2 activation on cardiac aging, it is important to acknowledge the dual role of ROS as both cytotoxic oxidants and essential intracellular signaling mediators. Excessive or prolonged antioxidant activation carries a theoretical risk of driving the redox state toward reductive stress, potentially interfering with physiological ROS-dependent signaling pathways. However, this scenario is unlikely in the present study, as aging is characterized by chronically elevated oxidative stress, a state in which reinforcing antioxidant defenses is expected to restore, not disrupt, redox homeostasis. In addition, while our findings imply extracellular vesicles as a plausible mechanism of inter-tissue communication, given the presence of antioxidant enzymes and related cargo in the systemic circulation, additional mechanistic studies are warranted. Future work should define the specific EV subtypes involved, characterize their proteomic and transcriptomic cargo with greater resolution, and delineate their uptake and downstream signaling pathways in cardiomyocytes. We are actively pursuing these directions, including isolating EVs from Nrf2-activated SkM and tracking their biodistribution and functional effects in cardiac cells both in vitro and in vivo. Finally, although our discussion highlights parallels between SkM Nrf2 activation and exercise-induced cardioprotection, these comparisons are conceptual, as no exercise group was included in the present study. The translational relevance of SkM-targeted Nrf2 activation in humans remains an emerging area, and while our findings provide mechanistic insight, practical application will require the development of safe, tissue-specific Nrf2 modulators and rigorous evaluation in clinical models of aging.

## 5. Conclusions

In conclusion, this study highlights the pivotal role of SkM Nrf2 activation in mitigating aging-induced cardiac dysfunction, providing new insights into the interorgan crosstalk mediated by redox homeostasis. By mimicking the molecular effects of chronic exercise, SkM Nrf2 activation offers a promising avenue for preserving cardiovascular health during aging. These findings open new avenues for research into antioxidant defense mechanisms and their therapeutic potential in age-related cardiac diseases. SkM Nrf2 activation can mitigate oxidative stress, inflammation, and mitochondrial dysfunction, thereby preserving cardiac structure and function during aging. The observed changes in redox and cardiac function proteins underscore the potential therapeutic implications of targeting Nrf2 for preventing sarcopenia and cardiac aging. Through proteomic analyses conducted on myocardial tissue, we explored the potential mechanisms underlying Nrf2-mediated cardioprotection. Previous research highlights the importance of Nrf2 in maintaining cellular redox balance and protecting against oxidative stress, both within skeletal muscle and systemically. Our findings suggest that Nrf2 activation may mitigate oxidative stress, inflammation, and mitochondrial dysfunction in the heart, thereby preserving cardiac structure and function during aging. The observed changes in redox and cardiac function proteins and pathways underscore the potential therapeutic implications of targeting SkM Nrf2 for preserving cardiac health in aging populations.

## Figures and Tables

**Table 1:** Significantly differentially expressed antioxidant proteins between A-WT and Y-WT and between A-KO and A-WT. Red, downregulated; blue, upregulated.

Gene	Description	logFC Aging-WT/Young-WT	logFC Aging-KO/Aging-WT
*Sod2*	Superoxide dismutase [Mn]	−0.7734859	0.8561775
*Glrx*	Glutaredoxin-1	−0.4202023	0.53331114
*Glrx3*	Glutaredoxin-3	−0.3643137	0.32566092
*Sod1*	Superoxide dismutase	−0.323255	0.10450807
*Txn2*	Thioredoxin, mitochondrial	−0.2258084	0.51128203
*Glrx5*	Glutaredoxin-5	−0.1030895	0.33220526
*Glrx2*	Glrx2 protein	−0.0253805	0.42047178

## Data Availability

The original contributions presented in this study are included in the article. Further inquiries can be directed to the corresponding authors.

## References

[1] Li H., Hastings M.H., Rhee J., Trager L.E., Roh J.D., Rosenzweig A. (2020). Targeting Age-Related Pathways in Heart Failure. Circ. Res..

[2] Sohal R.S., Orr W.C. (2012). The redox stress hypothesis of aging. Free Radic. Biol. Med..

[3] Ungvari Z., Tarantini S., Donato A.J., Galvan V., Csiszar A. (2018). Mechanisms of Vascular Aging. Circ. Res..

[4] Ji Z., Liu G.H., Qu J. (2025). Mitochondrial sirtuins, key regulators of aging. Life Med..

[5] Bellezza I., Giambanco I., Minelli A., Donato R. (2018). Nrf_2_-Keap_1_ signaling in oxidative and reductive stress. Biochim. Biophys. Acta Mol. Cell Res..

[6] Ma Q. (2013). Role of nrf_2_ in oxidative stress and toxicity. Annu. Rev. Pharmacol. Toxicol..

[7] Pomatto L.C.D., Davies K.J.A. (2018). Adaptive homeostasis and the free radical theory of ageing. Free Radic. Biol. Med..

[8] Pietrangelo D., Lopa C., Litterio M., Cotugno M., Rubattu S., Lombardi A. (2025). Metabolic Disturbances Involved in Cardiovascular Diseases: The Role of Mitochondrial Dysfunction, Altered Bioenergetics and Oxidative Stress. Int. J. Mol. Sci..

[9] Dhyani N., Tian C., Gao L., Rudebush T.L., Zucker I.H. (2024). Nrf_2_-Keap_1_ in Cardiovascular Disease: Which Is the Cart and Which the Horse?. Physiology.

[10] Ahn B., Pharaoh G., Premkumar P., Huseman K., Ranjit R., Kinter M., Szweda L., Kiss T., Fulop G., Tarantini S. (2018). Nrf_2_ deficiency exacerbates age-related contractile dysfunction and loss of skeletal muscle mass. Redox Biol..

[11] Fasipe B., Li S., Laher I. (2021). Harnessing the cardiovascular benefits of exercise: Are Nrf_2_ activators useful?. Sports Med. Health Sci..

[12] Li Q., Qian Z., Huang Y., Yang X., Yang J., Xiao N., Liang G., Zheang H., Fu Y., Lin Y. (2025). Mechanisms of endothelial senescence and vascular aging. Biogerontology.

[13] Gao L., Wang H.J., Tian C., Zucker I.H. (2021). Skeletal Muscle Nrf_2_ Contributes to Exercise-Evoked Systemic Antioxidant Defense Via Extracellular Vesicular Communication. Exerc. Sport Sci. Rev..

[14] Just J., Yan Y., Farup J., Sieljacks P., Sloth M., Veno M., Gu T., de Paoli F.V., Nyengaard J.R., Baek R. (2020). Blood flow-restricted resistance exercise alters the surface profile, miRNA cargo and functional impact of circulating extracellular vesicles. Sci. Rep..

[15] Gao L., Kumar V., Vellichirammal N.N., Park S.Y., Rudebush T.L., Yu L., Son W.M., Pekas E.J., Wafi A.M., Hong J. (2020). Functional, proteomic and bioinformatic analyses of Nrf_2_- and Keap_1_- null skeletal muscle. J. Physiol..

[16] Wafi A.M., Yu L., Gao L., Zucker I.H. (2019). Exercise training upregulates Nrf_2_ protein in the rostral ventrolateral medulla of mice with heart failure. J. Appl. Physiol..

[17] Ma A., Hong J., Shanks J., Rudebush T., Yu L., Hackfort B.T., Wang H., Zucker I.H., Gao L. (2019). Upregulating Nrf2 in the RVLM ameliorates sympatho-excitation in mice with chronic heart failure. Free Radic. Biol. Med..

[18] Kong X., Thimmulappa R., Craciun F., Harvey C., Singh A., Kombairaju P., Reddy S.P., Remick D., Biswal S. (2011). Enhancing Nrf_2_ pathway by disruption of Keap1 in myeloid leukocytes protects against sepsis. Am. J. Respir. Crit. Care Med..

[19] Walters T.S., McIntosh D.J., Ingram S.M., Tillery L., Motley E.D., Arinze I.J., Misra S. (2021). SUMO-Modification of Human Nrf_2_ at K^110^ and K^533^ Regulates Its Nucleocytoplasmic Localization, Stability and Transcriptional Activity. Cell. Physiol. Biochem..

[20] Lau A., Tian W., Whitman S.A., Zhang D.D. (2013). The predicted molecular weight of Nrf_2_: It is what it is not. Antioxid. Redox Signal.

[21] Kopacz A., Rojo A.I., Patibandla C., Lastra-Martinez D., Piechota-Polanczyk A., Kloska D., Jozkowicz A., Sutherland C., Cuadrado A., Grochot-Przeczek A. (2022). Overlooked and valuable facts to know in the NRF_2_/KEAP_1_ field. Free Radic. Biol. Med..

[22] Jyothidasan A., Sunny S., Murugesan S., Quiles J.M., Challa A.K., Dalley B., Cinghu S.K., Nanda V., Rajasekaran N.S. (2022). Transgenic Expression of Nrf_2_ Induces a Pro-Reductive Stress and Adaptive Cardiac Remodeling in the Mouse. Genes.

[23] Bhat A., Abu R., Jagadesan S., Vellichirammal N.N., Pendyala V.V., Yu L., Rudebush T.L., Guda C., Zucker I.H., Kumar V. (2023). Quantitative Proteomics Identifies Novel Nrf_2_-Mediated Adaptative Signaling Pathways in Skeletal Muscle Following Exercise Training. Antioxidants.

[24] Pribil Pardun S., Bhat A., Anderson C.P., Allen M.F., Bruening W., Jacob J., Pendyala V.V., Yu L., Bruett T., Zimmerman M. (2024). Electrical Pulse Stimulation Protects C_2_C_12_ Myotubes against Hydrogen Peroxide-Induced Cytotoxicity via Nrf_2_/Antioxidant Pathway. Antioxidants.

[25] Gao L., Zimmerman M.C., Biswal S., Zucker I.H. (2017). Selective Nrf2 Gene Deletion in the Rostral Ventrolateral Medulla Evokes Hypertension and Sympathoexcitation in Mice. Hypertension.

[26] Tian C., Gao L., Rudebush T.L., Yu L., Zucker I.H. (2022). Extracellular Vesicles Regulate Sympatho-Excitation by Nrf_2_ in Heart Failure. Circ. Res..

[27] Grotle A.K., Langlo J.V., Holsbrekken E., Stone A.J., Tanaka H., Fadel P.J. (2023). Age-related alterations in the cardiovascular responses to acute exercise in males and females: Role of the exercise pressor reflex. Front. Physiol..

[28] Bigalke J.A., Young B.E., Cleveland E.L., Fadel P.J., Carter J.R. (2024). Aging and sympathetic transduction to blood pressure in humans: Methodological and physiological considerations. Am. J. Physiol. Heart Circ. Physiol..

[29] Giunta S., Xia S., Pelliccioni G., Olivieri F. (2024). Autonomic nervous system imbalance during aging contributes to impair endogenous anti-inflammaging strategies. Geroscience.

[30] Rim D., Henderson L.A., Macefield V.G. (2022). Brain and cardiovascular-related changes are associated with aging, hypertension, and atrial fibrillation. Clin. Auton. Res..

[31] Frame A.A., Nist K.M., Kim K., Puleo F., Moreira J.D., Swaldi H., McKenna J., Wainford R.D. (2024). Integrated renal and sympathetic mechanisms underlying the development of sex- and age-dependent hypertension and the salt sensitivity of blood pressure. Geroscience.

[32] Tian C., Gao L., Zucker I.H. (2021). Regulation of Nrf_2_ signaling pathway in heart failure: Role of extracellular vesicles and non-coding RNAs. Free Radic. Biol. Med..

[33] Lin H., Yin L., Liu W., Li R., Jiang T., Yang M., Cao Y., Wang S., Yu Y., Chen C. (2025). Muscle-Derived Small Extracellular Vesicles Mediate Exercise-Induced Cognitive Protection in Chronic Cerebral Hypoperfusion. Adv. Sci..

[34] Ozerklig B., Turkel I., Yilmaz M., Vaizoglu R.D., Akan H.S., Dikmen Z.G., Saleem A., Kosar S.N. (2025). Exercise-induced extracellular vesicles mediate apoptosis in human colon cancer cells in an exercise intensity-dependent manner. Eur. J. Appl. Physiol..

[35] Lundy D.J., Liao C.T. (2025). Extracellular Vesicles in Aging and Age-Related Diseases. How Important Are They?. Adv. Biol..

[36] Obi P.O., Souza T.F.G., Ozerklig B., Seif S., Bydak B., Klassen N., Duhamel T.A., West A.R., Gordon J.W., Saleem A. (2025). Extracellular Vesicles Released from Skeletal Muscle Post-Chronic Contractile Activity Increase Mitochondrial Biogenesis in Recipient Myoblasts. J. Extracell. Vesicles.

[37] Allemann M.S., Lee P., Beer J.H., Saeedi Saravi S.S. (2023). Targeting the redox system for cardiovascular regeneration in aging. Aging Cell.

[38] Izzo C., Vitillo P., Di Pietro P., Visco V., Strianese A., Virtuoso N., Ciccarelli M., Galasso G., Carrizzo A., Vecchione C. (2021). The Role of Oxidative Stress in Cardiovascular Aging and Cardiovascular Diseases. Life.

[39] Morris B.J. (2013). Seven sirtuins for seven deadly diseases of aging. Free Radic. Biol. Med..

[40] Kitaoka Y. (2021). The Role of Nrf_2_ in Skeletal Muscle on Exercise Capacity. Antioxidants.

[41] Ngo V., Duennwald M.L. (2022). Nrf_2_ and Oxidative Stress: A General Overview of Mechanisms and Implications in Human Disease. Antioxidants.

[42] Zinovkin R.A., Grebenchikov O.A. (2020). Transcription Factor Nrf_2_ as a Potential Therapeutic Target for Prevention of Cytokine Storm in COVID-19 Patients. Biochemistry.

[43] Dinkova-Kostova A.T., Abramov A.Y. (2015). The emerging role of Nrf_2_ in mitochondrial function. Free Radic. Biol. Med..

[44] Bersell K., Choudhury S., Mollova M., Polizzotti B.D., Ganapathy B., Walsh S., Wadugu B., Arab S., Kuhn B. (2013). Moderate and high amounts of tamoxifen in αMHC-MerCreMer mice induce a DNA damage response, leading to heart failure and death. Dis. Model. Mech..

